# The Society Troubles in New York—A New Organization

**Published:** 1884-03-20

**Authors:** 


### *THE SOCIETY TROUBLES IN NEW YORK—A NEW ORGANIZATION.*

We have been kindly furnished with a pamphlet containing the minutes of a Convention of the friends of the National Code in New York, which assembled at Albany February 4th, 1884. The old State Association being in session at the same time and place, the new body made a final appeal to have the New Code, which had caused the division, abolished or repealed. It resulted in failure, notwithstanding proof was furnished, as shown in a canvass of the entire State, that a large majority of the Profession in the State was opposed to the New Code. The recorded votes, as taken throughout the State, are as follows: For the National Code, 2,549; for the New Code, 1040; for no code, 239; unclassified, 34. The above vote was from the State at large. Of these there were 382 upon the Society roll, the vote of whom was as follows: For the National Code, 193; for the New Code, 134: for no code, 19; uncommitted, 36. And yet, in the face of this showing, the Old Society refused to repeal its action. This led to the new organization, which assumes the name of *The New York State Medical Association.* The new body will hold its first meeting at New York City on the third Tuesday in November, 1884. The President elect is Dr. H. D. Didama, of Onandaga Co.; Recording Secretary, Dr. Caleb Green, of Courtland Co.; Corresponding Secretary, Dr. E D. Ferguson, Renssalaer Co.

